# Evaluating the attitude of medical students toward the impact of social media on improving learning and increasing awareness during the Covid‐19 pandemic: A cross‐sectional study in Iran

**DOI:** 10.1002/hsr2.1364

**Published:** 2023-06-20

**Authors:** Abdolreza Gilavand, Fakher Fakhri, Maryam Seyedtabib

**Affiliations:** ^1^ Department of Medical Education, School of Medicine Ahvaz Jundishapur University of Medical Sciences Ahvaz Iran; ^2^ Department of Community Medicine School of Medicine, Ahvaz Jundishapur University of Medical Sciences Ahvaz Iran; ^3^ Department of Biostatistics and Epidemiology, School of Health Ahvaz Jundishapur University of Medical Sciences Ahvaz Iran

**Keywords:** awareness, Covid‐19, learning, medical students, social media

## Abstract

**Introduction:**

The use of social media has become an inseparable part of many students' lives. Therefore, in this research, we have tried to evaluate the attitude of medical students toward the impact of social media on improving their learning and increasing their awareness during the coronavirus disease 2019 pandemic.

**Materials and Methods:**

This is a descriptive correlation study that was conducted in Iran in 2021. The statistical population of this study included medical students. Finally, 260 students participated in it. A researcher‐made questionnaire was used to collect data.

**Results:**

Most students use social media for educational purposes, including sending and sharing educational materials (42.8%), membership in study groups (42.6%), uploading educational content and presenting class topics (39.7%), chatting with students and professors (35.5%), formation of educational groups (34.2%), checking homework (32.7%), class discussion (30%), and sending and sharing photos and videos (29.5%). The most followed topics in social media by students to increase their level of awareness were respectively scientific topics not related to the field of study (46.9%), topics related to immigration (46%), cultural topics (44.2%), learning English (43.2%), academic and scientific materials related to the field of study (40.8%). Also, WhatsApp and Telegram were the most popular social media among students at the same time (58.8%). Also, students had a very positive attitude towards social media and considered them effective in improving learning and raising awareness.

**Conclusion:**

In general, the results of our study showed that social media could play an influential role in improving learning and raising the level of awareness of medical students. However, there may be problems and challenges in using these technologies, such as blocking the popular social media platform in some countries, the imposition of some restrictive government laws, low internet speed, protecting the privacy of students, professors, and so on.

## 1 INTRODUCTION

Today, a world with features created by computers and Internet communication has emerged, penetrating various areas of life and education. The revolution of information and communication technology has caused the prosperity of educational, economic, and cultural movements, and a new world is emerging. In this revolution, networks and information systems are considered the most important bases and channels of information transfer and exchange.^1^ Social media (SM) are referred to as online communities of Internet users who have the desire to communicate with other users in areas of mutual interest.^2^

The use of SM has become an inseparable part of many students' lives and has had a direct impact on all aspects of student life, including the amount of study time and academic performance.^3^, ^4^ However, using these media facilitates communication with friends. However, it may also disrupt their education by reducing the study time of students.^5^ However, SM does not have only adverse effects, and by using them in the educational or even therapeutic process, they can be optimally used for educational purposes.^6^ Recent studies have indicated that SM can be a valuable and cheerful resource for students in various aspects, provided they are used correctly.^5^

In Iran, about 38 years ago, the medical education system was integrated with the healthcare service delivery system. Medical students and their professors were engaged in teaching students and treating patients in teaching hospitals.^7^ The coronavirus disease 2019 (Covid‐19) pandemic negatively affected all aspects of people's lives worldwide, and the education and health sectors were no exception to this rule.^8^ Social distancing and mandatory quarantine confronted the university education system with obstacles and challenges. They forced the teaching and learning system from the face‐to‐face method to the online system and even affected the students' mental health.^9^ In addition to online education, SM such as WhatsApp, Telegram, LinkedIn, Instagram, Facebook, and so on also played an influential role in raising the level of students' awareness and improving their learning. In this research, we have tried to evaluate the attitude of medical students toward the impact of SM on improving their learning and increasing their awareness during the Covid‐19 pandemic.

## 2 MATERIALS AND METHODS

The current study is of applied type and is considered descriptive and correlational research. The statistical population of this study included all medical students of Ahvaz Jundishapur University of Medical Sciences (in the southwest of Iran) in 2021 (with at least 1 year of study experience in the field of medicine during the Covid‐19 pandemic). Based on the information obtained from similar studies, and considering the probability of type 1 error of 0.05 and accuracy of 0.05, the sample size was calculated to be around 195 students^10^; in total, 260 students participated in this research

$$p=\frac{pqz_{1-\frac{\alpha }{2}}^{2}}{d_{2}}.$$

The method of data collection in this research was in the form of completing a questionnaire, which was given to the students after obtaining their consent. The research questionnaire was prepared based on previous studies, especially the questionnaire made by Movahedi et al.^11^ This questionnaire had seven parts: questions 1–4, demographic characteristics; questions 5–8, skill questions; questions 9–12, the amount and type of students' activity in SM; questions 13–20, students' use of SM for learning and educational purposes; questions 21–29, topics followed in SM by students to increase their awareness; questions 30–42, the effects of SM on students' learning; and questions 61–43, the general attitude of students regarding SM. The validity and reliability of this questionnaire have been confirmed. To check the validity of the questionnaire, the opinions of faculty members and specialists in medical education and informatics of Ahvaz Jundishapur University of Medical Sciences were used. The electronic questionnaire was distributed among students using SM. The reliability of the questionnaire was also checked with Cronbach's α, which indicated the existence of convergence between the participants' responses and the same understanding of the content of the variables, which was 88%. Categorical data were reported as percentages, frequencies, mean, and standard deviation. The level of statistical significance was declared at *p* <  0.05. The statistical analysis was carried out using IBM's Statistical Package for the Social Sciences (SPSS) version 25.0 software.

## 3 RESULTS

Two hundred and sixty medical students participated in this study, of which 120 were women and 140 were men. Also, 95 of them were between 19 and 24 years old, 122 of them were between 25 and 34 years old, 30 of them were between 35 and 44 years old, and 13 of them were over 45 years old. Also, the students had stated the grade point average (GPA) of their passed courses (out of a maximum score of 20), 12 of them had a GPA of less than 12, 82 had a GPA between 12 and 14, 115 had a GPA between 15 and 16, 33 had a GPA between 17 and 18, and 18 of them declared their GPA between 19 and 20.

A total of 90.7% of students used personal mobile phones or tablets, 6.2% of students used personal computers or laptops, and 3.1% of students used university computers to access SM.

In Table 1, the skill level of the students was asked in four areas, which is given as a frequency (percentage), and in five categories from poor to excellent level. As can be seen, most of the answers are in the category of very good.

**Table 1:** Skill questions of students.

Items	Poor (%)	Fair (%)	Good (%)	Very good (%)	Excellent (%)
Level of skill—familiarity with the English language (in terms of writing)	26.8	17.7	21.2	26.2	8.1
Level of skill—familiarity with the English language (in terms of speaking)	23.8	16.9	28.1	20	11.2
How good are your computer skills?	5	20	31.2	31.9	11.9
How good are your internet skills?	2.7	15.4	31.9	35	15

Table 2 indicates the percentage of students who join SM to pursue academic and educational goals; as can be seen, the highest percentage is (simultaneously) related to WhatsApp and Telegram.

**Table 2:** Types of social media used by students to pursue academic and educational goals.

Variable	Classification	Frequency	Frequency (%)
To follow your learning and educational goals, which of the following social media are you a member of?	Academia	4	1.5
	Instagram	37	14.2
	Telegram	8	3.1
	Twitter	6	2.3
	Facebook	8	3.1
	LinkedIn	2	0.8
	WhatsApp	42	16.2
	WhatsApp and Telegram	153	58.8
	Total	260	100

Table 3 shows the number of hours students use SM during the weekdays and the hours they use SM the most. As can be seen, the most declared time is 4–6 h a day, and the most used time is from 6 p.m. to 12 midnight.

**Table 3:** Amount of use of social media during the days of the week.

Variable	Classification	Frequency	Frequency (%)
The amount of use of social media during the days of the week	1–2 h	17	6.5
	2–4 h	87	33.5
	4–6 h	109	41.9
	6–8 h	22	8.5
	More than 8 h	22	8.5
	Under an hour	3	1.2
What time do you use social media the most?	From 12 noon to 6 p.m.	78	30.0
	From 12 p.m. to 5 a.m.	34	13.1
	From 6 a.m. to 12 noon	18	6.9
	From 6 p.m. to 12 p.m.	105	40.4
	24 h a day	25	9.6
	Total	260	100

According to the results of Table 4, most students use SM for educational purposes, including, sending and sharing educational materials (42.8%), membership in study groups (42.6%), uploading educational content, and presenting class topics (39.7%), chat with students and professors (35.5%), formation of educational groups (34.2%), checking homework (32.7%), class discussion (30%), and sending and sharing photos and videos (29.5%).

**Table 4:** Students' use of social media for educational purposes.

Item	Too little	Little	At all	Much	Too much
Sending and sharing educational materials	19.2	4.2	33.8	6.5	36.3
Membership in study groups	19.0	4.4	34	7.0	35.6
Uploading educational content and presenting class topics	24.2	4.2	31.9	5.1	34.6
Chat with students and professors	32.7	5.1	26.7	5.4	30.1
Formation of educational groups	30	5.8	30.0	3.8	30.4
Checking homework	23.5	7.3	36.5	3.5	29.2
Class discussion	35.5	3.5	31.5	4.6	25.4
Sending and sharing photos and videos	36.2	3.5	30.8	3.2	26.3

According to Table 5, the most following topics on SM by students to increase their level of awareness were respectively scientific topics not related to the field of study (46.9%), topics related to immigration (46%), cultural topics (44.2%), learning English (43.2%), academic and scientific materials related to the field of study (40.8%), social contents (40.2%), economic contents (38.1%), political contents (30%), sports contents (24.2%) and just entertainment contents (12.4%).

**Table 5:** Following topics in social media by students to increase the level of awareness.

Item	Too little	Little	At all	Much	Too much
Scientific topics not related to the field of study	17.3	13.8	28.1	10.0	30.8
Topics related to immigration	20.4	14.6	18.1	11.5	35.4
Cultural topics	20.8	16.0	20.0	15.4	27.8
Learning English	20.4	13.5	28.0	6.2	31.9
Academic and scientific materials related to the field of study	20.8	15.0	20.0	16.9	27.3
Social contents	19.6	21.5	18.7	16.6	23.6
Economic contents	33.5	2.3	34.2	3.5	26.5
Political contents	31.2	16.0	28.6	1.5	22.7
Sports content	40.0	15.2	32.4	2.4	10.0
Just entertainment contents	12.0	14.0	28.0	10.0	36.0

In Table 6, the attitude of medical students toward SM is given, which shows the positive attitude of students regarding the use of SM to improve learning and raise the level of their awareness.

**Table 6:** Attitude of medical students toward social media.

Item	Strongly disagree	Disagree	Neither agree nor disagree	Agree	Strongly agree
Social media can be used in research	3.8	5.8	29.2	28.5	32.7
Using social media requires a cultural link	1.2	4.2	18.5	36.2	40
The cost of social media is less compared to other information sources	1.9	2.7	21.2	40.8	33.5
Social media make students excited during education	1.2	5.0	21.5	29.2	43.1
Belief in the usefulness of social media affects its use	0.8	2.3	21.9	38.5	36.5
Social media can be a tool for getting to know international experts	2.3	4.2	30.8	28.8	33.8
Social media are an excellent tool to access the latest scientific findings	2.7	6.9	30.8	36.5	23.1
Social media are an essential tool for knowing about international conferences and conventions	1.5	2.7	18.8	33.8	43.1
Using social media in educational and research activities does not conflict with using other sources	0.8	1.5	18.1	40.4	39.2
Social media simplify access to information needed for research activities	1.2	4.2	26.9	30.4	37.3
Using social media helps to improve the level of English knowledge	1.2	4.6	28.1	26.5	39.6
Information on social media is more up‐to‐date compared to printed sources	2.7	6.9	30.8	36.5	23.1
By using social media, you can reach different types of content faster	1.5	2.7	18.8	33.8	43.1
Much information can be obtained only through social media	0.8	1.5	18.1	40.4	39.2
In research, it is easy to compare the advantages of social media with printed sources	1.2	4.2	26.9	30.4	37.3
The quality of education increases through the use of social media	1.2	4.6	28.1	26.5	39.6
Social media are a suitable tool for transferring educational content	3.2	2.6	28.1	26.5	39.6
Using social media increases and develops professional activities	1.5	2.7	18.8	33.8	43.1
Social media make the learning process easier	3.7	5.9	30.8	36.5	23.1
Using social media increases interest in education	2.7	6.9	30.8	36.5	23.1
Social media cause creativity	2.4	4.2	30.8	28.8	33.8
Public use of social media is an essential factor in encouraging people to use them	1.0	1.3	18.0	40.5	39.2
Social media can make education faster	2.0	2.7	20.1	38	37.2
Social media are a good tool for chatting and communicating with friends	4.8	4.8	30.2	27.5	32.7
Social media can offer more job opportunities to users	5.5	3.6	21.5	29.4	40.0
Social media users can provide people with the necessary information to choose a job	1.4	2.8	18.6	36.2	41.0
Using social media does not conflict with national and religious values	6.0	2.6	22.0	3.4	39.0

In Table 7, the relationship between gender and skill areas, use for learning and educational purposes, examining the effects of SM, attitude, and raising the level of awareness was measured using the Mann–Whitney test; there was a significant relationship between skill areas and use for learning and educational purposes and gender so that men had obtained a higher score in the skill areas and use for learning and educational purposes.

**Table 7:** Relationship between the gender variable and the areas of the questionnaire.

Areas	Gender	mean	SD	*p* Value
Skill	Women	11.57	3.45	0.01
	Men	12.65	3.22	
Use for learning and educational purposes	Women	25.47	9.90	0.01
	Men	30.71	8.58	
Investigating the effects of social media	Women	73.80	12.14	0.60
	Men	74.60	12.86	
Attitude	Women	103.64	17.22	0.24
	Men	106.33	19.48	
Raising the level of awareness	Women	31.22	4.93	0.72
	Men	31.46	5.61	

In Table 8, the relationship between the variable of the student's age and the skill areas, use for learning and educational purposes, investigating the effects of SM, attitude, and raising the level of awareness was measured using the Kruskal‐Wallis test; there was only a significant relationship between the variable of age and the area of attitude so that students aged between 19 and 24 and also between 35 and 44 had a better attitude toward SM.

**Table 8:** Relationship between the variable of age and the areas of the questionnaire.

independent variable	Dependent variable	Mean	SD	*p* Value
Skill	Over 45 years old	31.53	5.39	0.41
	Between 25 and 34 years	31.20	4.98	
	Between 19 and 24 years	31.77	5.11	
	Between 35 and 44 years	31.13	5.48	
Use for learning and educational purposes	Over 45 years old	103.58	21.08	0.25
	Between 25 and 34 years	103.46	18.69	
	Between 19 and 24 years	107.77	16.56	
	Between 35 and 44 years	102.10	19.82	
Investigating the effects of social media	Over 45 years old	74.63	13.76	0.49
	Between 25 and 34 years	73.20	13.75	
	Between 19 and 24 years	75.37	11.92	
	Between 35 and 44 years	74.23	12.08	
Attitude	Over 45 years old	26.68	9.38	0.001
	Between 25 and 34 years	24.34	10.13	
	Between 19 and 24 years	30.80	9.22	
	Between 35 and 44 years	29.57	7.01	
Raising the level of awareness	Over 45 years old	10.63	3.61	0.11
	Between 25 and 34 years	12.11	2.65	
	Between 19 and 24 years	12.13	3.20	
	Between 35 and 44 years	12.67	4.06	

## 4 DISCUSSION

Our study showed that medical students have a very positive attitude toward SM and consider them effective in improving learning and raising awareness. Also, WhatsApp and Telegram were the most popular SM among students for learning purposes.

Kimiafar et al.^10^ conducted a study on the benefits and barriers of SM for learning purposes among medical students in Northeast Iran; most of the students were inclined to use SM for learning purposes. In this study, sending and receiving educational videos and sending and receiving educational texts, posts, and materials were the most important for students using SM.

Javadinia et al.^12^ in research entitled the effect of using virtual SM on the academic performance of the students of the University of Medical Sciences in Northeast Iran concluded that using SM can lead to the student's academic progress; however, using this technology should be introduced to students at the beginning of their entry into the university.

The findings of Malmir et al.'s research in the west of Iran showed that the level of academic progress and memorization of students who learned English through virtual SM was higher than the level of academic progress and memorization of students who have been traditionally taught English.^13^

Wanner et al.'s study showed that many medical students use SM to enhance their education. Also, most students support the integrating SM into their education. They suggested that educators formally incorporate SM into curricula designed for medical students to augment traditional methods such as textbooks and lectures.^14^

Alsuraihi et al., in their study on the use of SM in medical education in Saudi Arabia, also showed that 95.8% of medical students found social networks helpful and were willing to use them in education. Also, 87.7% said they use SM for educational purposes.^15^

The results of Latif and colleagues research in Pakistan showed that SM have a high potential in medical education and provide students with an opportunity to participate, share, and express knowledge and information with each other. In this research, Facebook, WhatsApp, and Edmodo were the most used.^16^

Cachón‐Pérez et al.'s study showed that using SM helped students feel closer to their professors and presented it as an opportunity to address a possible lack of correlation between theory and practice. In addition, SM helped them create an image of nursing in clinical settings. By using SM, nursing students could integrate and apply the knowledge acquired at university during their clinical courses in hospitals.^17^

Lima et al. conducted a systematic review study entitled “Social media as a tool for surgical education, in which 24 eligible articles were reviewed, among which 11 articles focused on laparoscopic surgery, 1 article on robotic‐assisted surgery, 1 article on cardiac surgery, and 11 articles on the specialty of general surgery (regardless of surgical approach or technique).^18^

The results of Benetoli et al.'s review study showed that social networks had been used as an educational tool in pharmacy education with positive feedback from students.^19^

The results of these studies are consistent with those of our study.

Our study showed that WhatsApp and Telegram are the most popular and widely used SM in improving the learning of students and raising the level of their awareness.

Coleman et al. conducted a systematic review study to investigate the role of WhatsApp in medical education, in which seven studies with 647 participants reported improvement in learners' knowledge after learning through WhatsApp.^20^ In a review study, Martins et al. reviewed nine studies published between 2016 and 2020 that used WhatsApp in dental education to facilitate communication and improve learning.^21^ Iqbal et al. conducted a study to investigate the role of Telegram as a tool to complete online medical education during the Covid‐19 pandemic. This study showed that Telegram provided an effective learning platform for medical students during the Covid‐19 pandemic. Also, the researchers announced that Telegram had better functions and fewer potential disadvantages than alternative SM applications.^22^

The results of Amiri et al.'s study in Iran showed that medical students used SM the most to obtain health‐related information, and these media have a significant effect on their willingness to perform preventive behaviors during the Covid‐19 pandemic.^23^

The results of these studies are also consistent with those of our study.

Despite some advantages of using SM in education and learning, the adverse effects, challenges, and obstacles of its use should not be neglected. Spending too much time using social networks may hurt the normal process of students' education and disrupt their academic and academic performance; in other words, addiction to SM will affect other aspects of students that can reduce their academic progress.

Emamirizi conducted research entitled the effect of virtual SM on the academic progress of female students in Western Iran. This study showed that the excessive use of these networks and spending too much time on these networks reduces students' study time. Also, since most students spend long hours of the night on these networks, they cannot attend classes with enough concentration (mainly due to lack of sleep), and these factors can lead to a decrease in their academic progress.^5^

Kirschner and Karpinski,^4^ in their study on the effect of SM on the student's academic performance in the Netherlands, stated that students who use Facebook and Twitter spend fewer hours studying university courses and obtain a lower overall GPA.

Many countries have published policies regarding the professional use of SM, such as the American Medical Association's policy.^21^ In Iran, the government has no favorable opinion or view toward non‐Iranian SM. Facebook, YouTube, and Twitter were already blocked. Recently, Telegram and then WhatsApp, which are very popular among students, have been blocked, and it is recommended to use Iranian SM. However, most students are still using them with the help of Proxy and VPN.^24^

## 5 LIMITATIONS

The statistical population of our study was only medical students. Therefore, the sample was not representative of SM users in general, which may limit generalizability to other populations.

## 6 CONCLUSION

In general, the results of our study showed that SM could play an influential role in improving learning and raising the level of awareness of medical students, and can provide various opportunities for student's education. However, there may be problems and challenges in using these technologies, such as blocking the popular SM platform in some countries, the imposition of some restrictive government laws, low internet speed, protecting the privacy of students and professors, and so on.

## AUTHOR CONTRIBUTIONS

**Abdolreza Gilavand**: Conceptualization; data curation; funding acquisition; methodology; project administration; supervision; writing—original draft. **Fakher Fakhri**: Formal analysis; validation; visualization. **Maryam Seyedtabib**: Writing—review and editing.

## CONFLICT OF INTEREST STATEMENT

The authors declare no conflict of interest.

## ETHICS STATEMENT

This research was derived from a research project (Ethics Code of IR.AJUMS.MEDICINE.REC.1400.066); therefore, have been ultimately observed by the author.

## TRANSPARENCY STATEMENT

The lead author Abdolreza Gilavand affirms that this manuscript is an honest, accurate, and transparent account of the study being reported; that no important aspects of the study have been omitted; and that any discrepancies from the study as planned (and, if relevant, registered) have been explained.

## Data Availability

The original contributions presented in the study are included in the article/Supporting Information: Material. Further inquiries can be directed to the corresponding author.
